# Discovery and synthesis of leaderless bacteriocins from the Actinomycetota

**DOI:** 10.1128/jb.00298-24

**Published:** 2024-10-15

**Authors:** David Hourigan, Felipe Miceli de Farias, Paula M. O’Connor, Colin Hill, R. Paul Ross

**Affiliations:** 1 APC Microbiome Ireland, University College Cork, Cork, Ireland; 2 School of Microbiology, University College Cork, Cork, Ireland; 3 Teagasc Food Research Centre, Cork, Ireland

**Keywords:** bacteriocins, Actinomycetota, leaderless bacteriocin, aureocin A53, antimicrobial-peptide

## Abstract

Leaderless bacteriocins are a unique class of bacteriocins that possess antimicrobial activity after translation and have few cases of documented resistance. Aureocin A53 and lacticin Q are considered two of the most well-studied leaderless bacteriocins. Here, we used *in silico* genome mining to search for novel aureocin A53-like leaderless bacteriocins in GenBank and MGnify. We identified 757 core peptides across 430 genomes with 75 species found currently without characterized leaderless bacteriocin production. These include putative novel species containing bacteriocin gene clusters (BGCs) from the genera *Streptomyces* (sp. NBC_00237) and *Agrococcus* (sp. SL85). To date, all characterized leaderless bacteriocins have been found within the phylum Bacillota, but this study identified 97 core peptides within the phylum Actinomycetota. Members of this phylum are traditionally associated with the production of antibiotics, such is the case with the genus *Streptomyces*. Actinomycetota is an under-explored phylum in terms of bacteriocin production with no characterized leader-less bacteriocin production to date. The two novel leaderless bacteriocins arcanocin and arachnicin from Actinomycetota members *Arcanobacterium* sp. and *Arachnia* sp., respectively, were chemically synthesized and antimicrobial activity was verified. These peptides were encoded in human gut (PRJNA485056) and oral (PRJEB43277)-microbiomes, respectively. This research highlights the biosynthetic potential of Actinomycetota in terms of leaderless bacteriocin production and describes the first antimicrobial peptides encoded in the genera *Arcanobacterium* and *Arachnia*.

Bacteriocins are ribosomally synthesized antimicrobial peptides/proteins that often have antimicrobial activity against strains similar to the producing organism (1). It is estimated that approximately 50% of all bacteria have the genetic capacity to produce at least one bacteriocin (1). They can be broadly categorized into two main classes: class 1 is ribosomally produced and post-translationally modified peptides (RiPPs), and class 2 is unmodified peptides (2). RiPPs undergo a series of modifications and require extracellular transport and regulation of production, which usually results in gene clusters above 10 kb in size. Class 2 has less complex biosynthetic gene clusters (BGCs) and do not undergo post-translational modifications. Recent advancements with *in silico* detection methods of bacteriocin BGCs across expanding databases have led to the detection of thousands of novel potential bacteriocins that could have antimicrobial activity against etiological causes for concern, such as the case with lanthipeptides, thiopeptides, and circular bacteriocins (3–5). Deep sequencing and assembly of metagenome-assembled genomes (MAGs) can uncover the genomic content of thousands of unculturable bacteria and have facilitated the in-depth study of microbiomes and their functional potential (6). This abundance of microbial genomes is frequently subjected to bioinformatic mining to decipher the biosynthetic potential of microbiomes and uncover mechanisms of competition and communication between bacteria (7, 8). However, detecting these potentially bioactive peptides has not coincided with advancements in production or the ability to culture obscure bacteria encoding novel gene clusters.

Leaderless bacteriocins represent a medically and commercially attractive subclass of these peptides due to the ability to genetically engineer, synthesize, and hence mass produce them. Indeed, the lack of a leader sequence to direct peptide export renders these peptides unusual; thus, they are active immediately after translation, meaning the producing organism is susceptible. This can be seen with lacticin Q, which can be intracellularly toxic (9). This peculiar lack of identifiable mechanism of leading the peptide for extracellular transport and known ligands means that leaderless bacteriocins remain somewhat of an enigma. Canonical aureocin A53-like leaderless bacteriocins have a characteristic structure similar to circular bacteriocins in that they possess four to five helices that conformationally form a small globular structure. However, a key differentiating factor is that circular bacteriocins are N–C terminal covalently linked, whereas leaderless bacteriocins are linear (10, 11). Leaderless bacteriocins have a formylated methionine at the N-terminal, which is not crucial for antimicrobial activity (10). To date, over 21 leaderless bacteriocins have been described in the literature, including aureocin A53 which is considered to be the prototypical leaderless bacteriocin that defines the class (12). Non-aureocin-like leaderless bacteriocins have been found that are shorter in length and have fewer tryptophan residues in their core peptides (13, 14). Aureocin A53 is a 51 residue, 6 kDa, highly cationic peptide with five tryptophan residues (12). These tryptophan residues contribute to the stability of the peptide and its mechanism of action and play a role in both oligomerization and protease resistance (15). The BGC in *Staphylococcus aureus* A53 is on the 10.4-kb plasmid pRJ9 (12). Interestingly, the operon produces the peptide without obvious regulatory elements or a protease. However, ABC transporter-like machinery has been described (16). Aureocin A53 has activity against *Listeria monocytogenes, S. aureus*, and *Streptococcus agalactiae* and is gamma-hemolytic against sheep blood (17–19). It has a broad spectrum of activity against gram-positive bacteria due to a mechanism of action that involves destabiliz ing the bacterial membrane. It has potent activity against clinically-relevant pathogens such as vancomycin-resistant enterococci (VRE) [minimum inhibitory concentration (MIC) 0.29 μg/mL] (17). Recently discovered leaderless bacteriocins include bawcin, a 48 amino acid-long peptide produced by *Bacillus wiedmannii* and toyoncin, a 70 amino acid-long peptide produced by *Bacillus toyonensis* XIN-YC13 (20, 21). Leaderless bacteriocins can be broadly categorized based on the number of core peptides they possess in their BGCs, that is, single peptide bacteriocins, two peptide bacteriocins, and multi-peptide bacteriocins (13, 14). However, leaderless bacteriocins can also be further categorized based on amino acid sequence into aureocin A53-like peptides, lsbB-like peptides, and entL-50-like peptides (13, 14).

Advancements in *in silico* detection methods of BGCs in tandem with the impressive increase of available genomes for mining have led to the expansion of modified class 1 bacteriocin BGCs to the thousands (3, 4). These methods have exploited the modification machinery of RiPPs to identify novel core peptides. A recent study has expanded this search to circular bacteriocins and expanded the repertoire of peptides to over 6,000 non-unique peptides (5). A recent study identified the leaderless bacteriocin miticin through genome mining using core peptides as driver sequences (22). This novel leaderless bacteriocin was encoded in a *Streptococcus mitis* genome and has activity against *Streptococcus pyogenes, S. aureus, Lactococcus lactis*, and *L. monocytogenes*. However, in uncovering how widespread bacteriocin production is across the bacterial kingdom, current literature on characterized bacteriocins is skewed toward the phylum Bacillota. There is a paucity of knowledge from the phylum Actinomycetota related to bacteriocin production considering the role they have played in the production of traditional antibiotics (23, 24). The phylum is high-GC content and encompasses the genera *Bifidobacterium, Curtobacterium, Arcanobacterium, Actinomyces*, and notably, *Streptomyces*, which is considered one of the most bioactive genera in terms of antimicrobial production (25).

To our knowledge, this study is the first in-depth systematic mining study to identify novel aureocin A53-like peptides and characterize how widespread they are among bacteria by searching the non-redundant (nr) database and MAGs through MGnify (26). MGnify is a microbiome data resource with over 2.4 billion nr sequences from MAGs (26). Here, we identified 757 putative peptides in 430 genomes and synthesized two novel peptides, arachnicin from *Arachnia* sp. and arcanocin from *Arcanobacterium* sp. both from the phylum Actinomycetota. We also highlight that the Actinomycetota are an underexplored reservoir of leaderless bacteriocins with 97 peptides discovered within the phylum. The synthesized peptides, arcanocin and arachnicin from the *Arcanobacterium* and *Arachnia* genera, respectively, are also the first leaderless bacteriocins reconstructed from metagenomic data sets and the first described from within the Actinomycetota.

## Results

### Phylogenetic distribution and biochemical properties of leaderless bacteriocins

**Fig 1:**
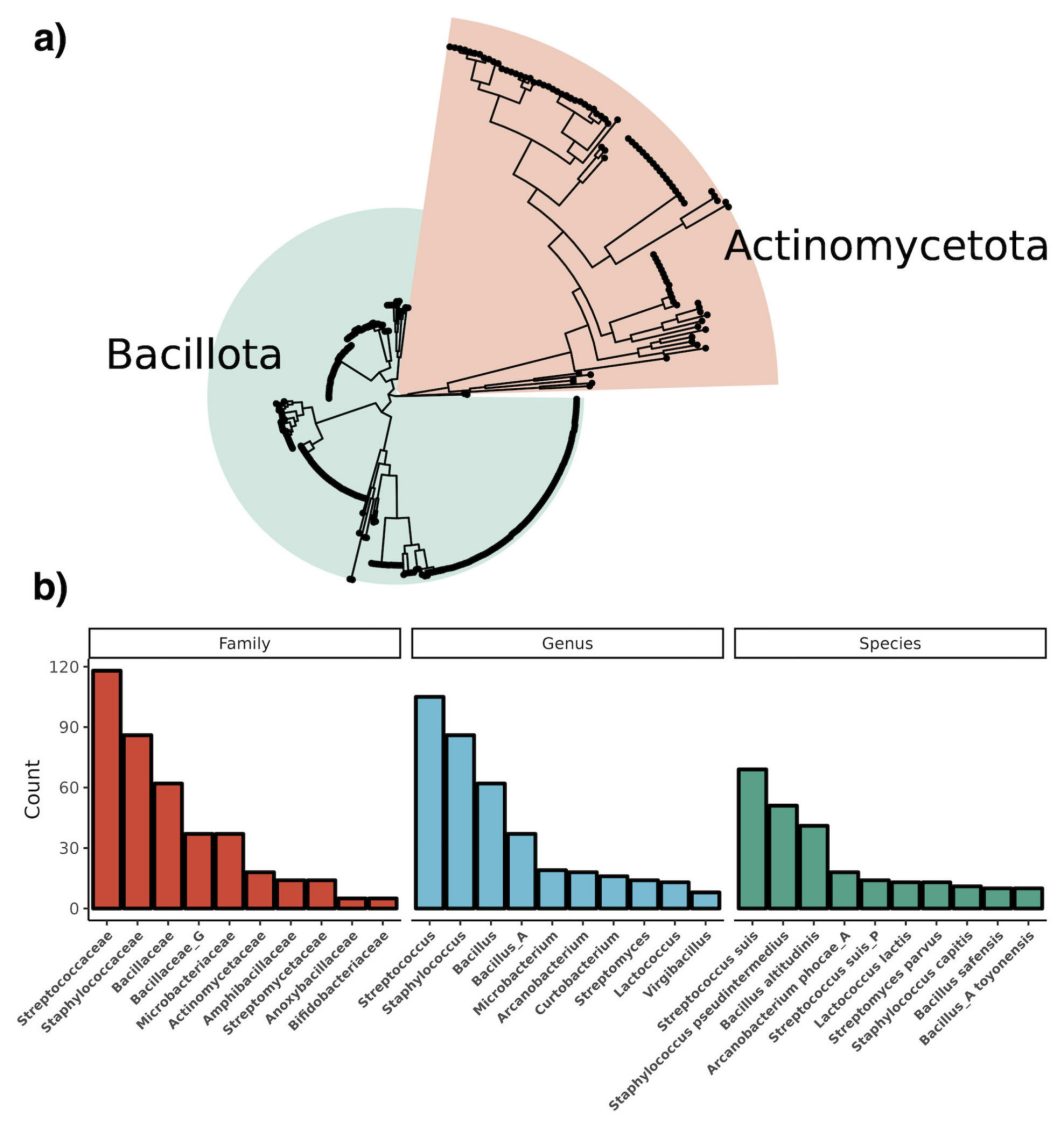
Leaderless bacteriocins are found widespread in the phyla Bacillota and Actinomycetota. (a) A phylogenetic tree of WGS of strains encoding leaderless bacteriocins. The phylogenetic tree was constructed using GTDB-Tk using shared marker genes and rooted to *Exiguobacterium* sp. s191 (GCF_018618955.1). The phylum Actinomycetota makes up 21% of genomes encoding a BGC. (b) A bar chart shows the top 10 species, genera, and families with putative leaderless bacteriocins. The family Streptococcaceae is dominated by *S. suis* genomes, and *A. phocae* is the species with the most core peptides within the phylum Actinomycetota.

**Fig 2:**
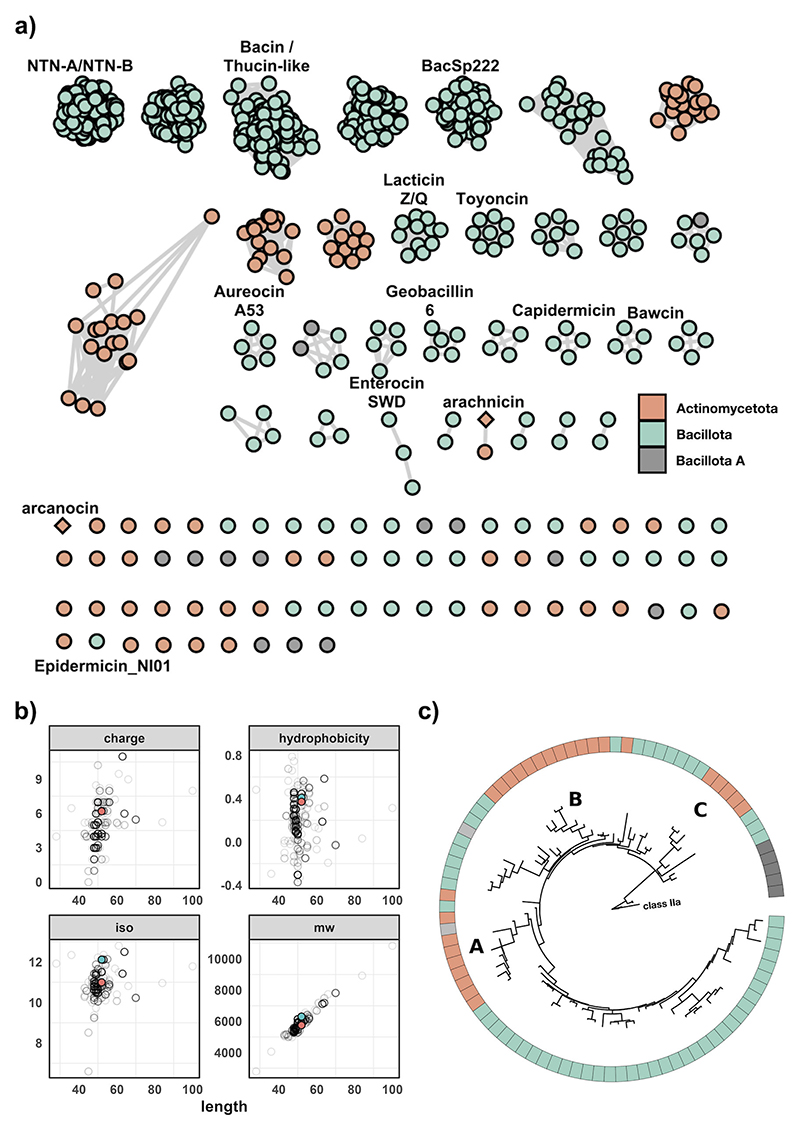
Insights into the diversity and physiochemical properties of Aureocin A53-like bacteriocins. (a) A SSN of leaderless bacteriocins. Orange represents leaderless bacteriocins in the phylum Actinomycetota, and green represents sequences in the phyla Bacillota. A diamond shape represents protein sequences that were synthesized. The singleton diamond is from *Arachnia*, and the non-singleton is from *Arcanobacterium*. (b) The physiological properties of all 757 leaderless bacteriocins, highlighted in green and orange, are peptides from *Arachnia* and *Arcanobacterium*, respectively, which were brought forward for peptide synthesis. Isoelectric point is labelled “iso,” molecular weight in daltons is labelled “mw.” (c) A reduced phylogenetic tree of all leaderless sequences clustered at 90% ID with a single representative for each cluster. The tree results from 1,000 bootstrap replicates, and each core peptide is colored by the genome’s phyla containing the representative sequence in which the cluster was found. The tree is rooted to pediocin PA-1.

Using a custom aureocin A53-like Hidden Markov Model (HMM) to screen the nr and MGnify databases, a total of 757 genes encoding putative leaderless bacteriocin core peptides were predicted across 95 unique species. A total of 430 genomes contained these genes. These were split between the phyla Actinomycetota (89/430; 20.7%), Bacillota (334/430; 77.7%), and Bacillota_A (7/430; 1.6%) (Fig. 1a). There are three putative novel species containing BGCs from the genera *Streptomyces* (sp. NBC_00237), *Agrococcus* (sp. SL85), and *Actinoplanes* (sp. NEAU-A12) as determined by the Genome Taxonomy Database (GTDB). A total of 322 BGCs were found encoding a single core peptide, while BGCs with multiple leaderless bacteriocins in operons were mainly within the genus *Bacillus*, with 52 BGCs with four or more core peptides. Genomes encoding six or more core peptides were almost exclusively in the genus *Bacillus*, apart from a single operon found in *Exiguobacterium* *indicum* containing seven core peptides (Table S1). In total, there are 75 species not currently known to produce leaderless bacteriocins, suggesting that their production is more widespread than previously thought. Some of these species include strict anaerobic bacteria within the class Clostridia and potential pathogens of etiological concern, such as *Streptococcus suis* and *Bacillus anthracis*. A total of 69 peptides were identified in *S. suis*, 51 in *Staphylococcus pseudintermedius*, 41 in *Bacillus altitudinis*, and 18 in *Arcanobacterium phocae*, which is the species within the Actinomycetota with the most core peptides (Fig. 1b). Streptococcaceae has the most BGCs, dominated by polyphyletic *S. suis* and *S. suis P* species. Of note is the existence of singleton peptides among the genera *Curtobacterium, Microbacterium*, and *Frondihabitans*, which are among multiple genera that have no described bacteriocins of any nature to date. Predicted peptides have a mean length of 50, a mean hydrophobicity of 0.16, and a mean charge of 4.63. An interesting note is that core peptides from the Actinomycetota are, on average, longer, more hydrophobic, and more positively charged as compared to peptides from the Bacillota (Fig. S1). In order to organize the diverse array of peptides, they were clustered using BLAST all-vs-all, which resulted in 31 groups (Fig. 2a). Singletons represent novel leaderless bacteriocins with unique amino acid sequences with <64.29% ID to another peptide, and the network within cluster percent identity is more than 64.29%. The majority of BGCs contained a single core peptide in their operon, and this was the case for both Actinomycetota and Bacillota. The majority of multi-peptide leaderless bacteriocin operons were among the Bacillota, with only eight assemblies having more than one core peptide within the Actinomycetota (Table S1). A total of 46 assemblies had more than four core peptides in their genome with instances of eight core peptides in a single gene cluster, as is the case with *Bacillus mycoides* that encode five proteins with 85.4% ID to thucin A1 (GCF_018739365.1). The operon lies within a putative IS6 family transposon and contains four separate, distinct core peptides with five copies of a single peptide. Another multi-component bacteriocin exists in *E. indicum* with core peptides ranging from 59.6% ID to thucin A2 and 31.25% ID to geobacillin 6 (GCF_018618955). The gene cluster is on a contig edge and likely contains another core peptide as there is another core peptide in the genome on a separate contig edge. This represents a genus without a described bacteriocin to date, and the gene clusters encode a Pleckstrin homology (PH) domain-containing protein and a helix-turn-helix domain-containing protein, suggesting a functionally-regulated operon. Of the eight leaderless bacteriocin operons with multiple core peptides within the phylum Actinomcyetota, seven are among the genus *Microbacterium* and a single genome from the species *Brevilactibacter sinopodophylli*, synonym *Propioniciclava* *sinopodophylli* with a core peptide 41.7% ID to thucin A1 (Table S1). *Brevilacibacter* *flavus* is a recently described novel genus with no characterized antimicrobial activity (27). All core peptides were clustered at 90% ID. A member of each cluster was used to construct a phylogenetic tree rooted to the class 2a bacteriocin pediocin PA-1 (Fig. 2c). Three distinct clades of leaderless peptides can be seen within Actinomycetota, which are designated A, B, and C. Clade A represents peptides most similar to NTN-A/B with the representative of the clade being a core peptide from *Streptomyces parvus* having <40% ID to NTN-A. Clade B is most similar to thucin-like peptides, and the representative core peptide from a *Paenarthrobacter* sp. has 43.75% ID to thucin A1. A core peptide from *Microbacterium* sp. with 33.33% ID to geobacillin 6 represents clade C.

### Leaderless bacteriocin BGCs of interest

**Fig 3:**
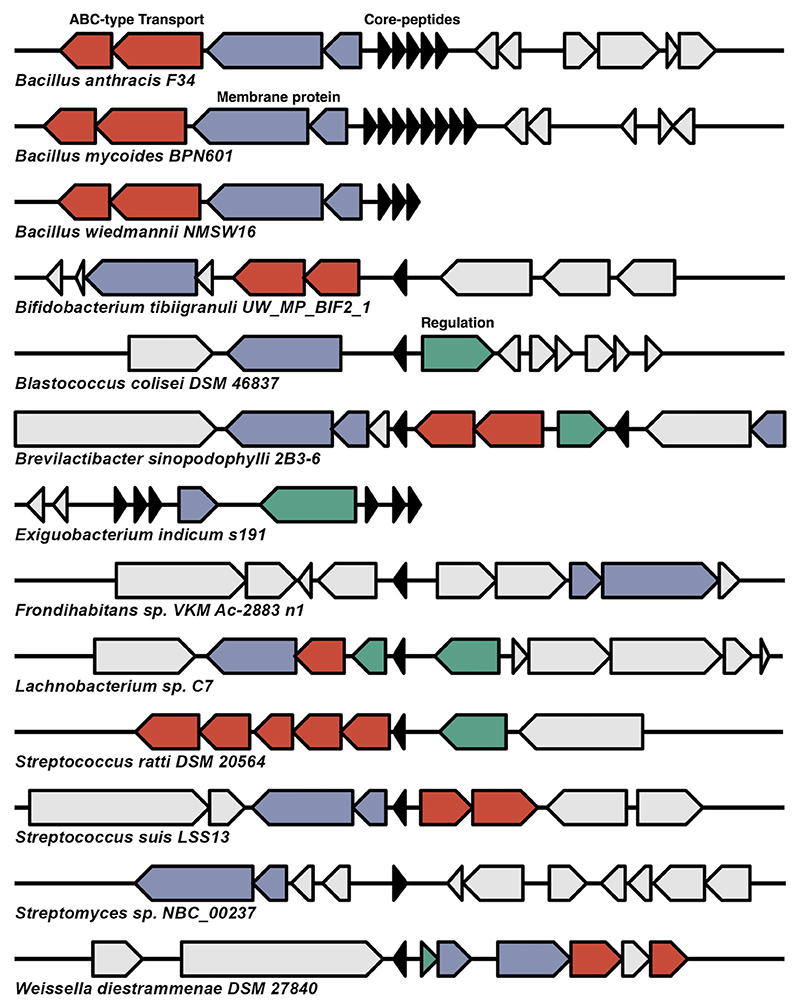
Operons of BGCs from assemblies deemed of particular interest. The plot depicts 13 operons from species of particular interest based on the number of core peptides present or if the operon is from a species without characterized leaderless bacteriocin operons. *Bifidobacterium, Blastococcus, Brevilactibacter, Exiguobacterium, Frondhabitans*, and *Lachnobacterium* are all genera without characterized bacteriocin production to date. Red arrows depict proteins involved in transport with an ABC transport-associated domain. Blue arrows encode membrane domain proteins such as Yip1 and PH domain-containing proteins. Green arrows are proteins involved in operon regulation, and black arrows are core peptides.

*B. mycoides* has an operon encoding eight core peptides and a shared synteny between *B. anthracis* and *B. wiedmannii. B. wiedmannii* is a known producer of the leaderless bacteriocin bawcin, and *B. anthracis* can be a producer of anthrax toxin, but this isolate is a non-toxigenic isolate (GCF_002024565.1). These operons share a high degree of synteny, with all three having multiple membrane-anchored proteins, including a PH domain-containing protein and ABC transport systems that consist of ABC-type lipoprotein export system multi-drug efflux pump subunit AcrA (membrane fusion protein). All three BGCs encode multiple core peptides (Fig. 3). *B. mycoides* encodes four distinct peptides with five gene copies encoding a single peptide. The five same encoded peptides are 85.4% ID to thucin A1 from *Bacillus thuringiensis* P86, and the most dissimilar is a single peptide with 60.4% to thucin A1. The *Bacillus* core peptides are shared between species (Fig. S1). *S. suis* encodes a single core peptide with 40.0% ID to lacticin Q, and the same peptide is encoded in a ST1 *S. suis* isolate from a diseased pig from the United Kingdom (GCF_000944375.1). *Lachnobacterium* sp. C7 (GCF_900113385.1) is a strict gut anaerobic bacterial isolate encoding a single core peptide with 48.8% ID to mutacin BHT-B (Fig. 3). The gene cluster encodes a putatively entire functional operon with an ABC-2 type transport system, an integral membrane protein similar to a PH domain-containing protein with putative function involved in the transport of the bacteriocin and a LacI family transcriptional regulator. *Bifidobacterium tibiigranuli* is a high-quality MAG encoding a core peptide 49.1% ID to lacticin Q from *L. lactis*. The operon encodes a membrane-associated PH domain and a dedicated ABC transport system. *Blastococcus colisei* BMG 822^T^ is a slow-growing member of the family *Geodermatophilaceae* isolated from an archaeological amphitheatre in Tunisia, and the genus has no characterized bacteriocin production (28). The isolate encodes a core peptide with 46% ID to lacticin Q, a PH domain-containing protein, a transcriptional regulator protein and is located less than 2 kb from a site-specific integrase (Fig. 3.). *Brevilactibacter* *sinopodophylli* (GCF_947488915.1) is a bacterium isolated from the phycosphere of marine macroalgae. It is within the family *Propionibacteriaceae* and the only member of the genus *Brevilactibacter* with a predicted BGC in this study. The strain encodes two distinct core peptides with 41.7% ID to thucin A1 and 39.6% to lacticin Q. The operon also encodes a PH domain-containing protein, an ABC transport system, and a DNA-binding response regulator, suggesting a functional operon.

### Novel aureocin A53-like bacteriocins from *Arachnia* and *Arcanobacterium*

**Fig 4:**
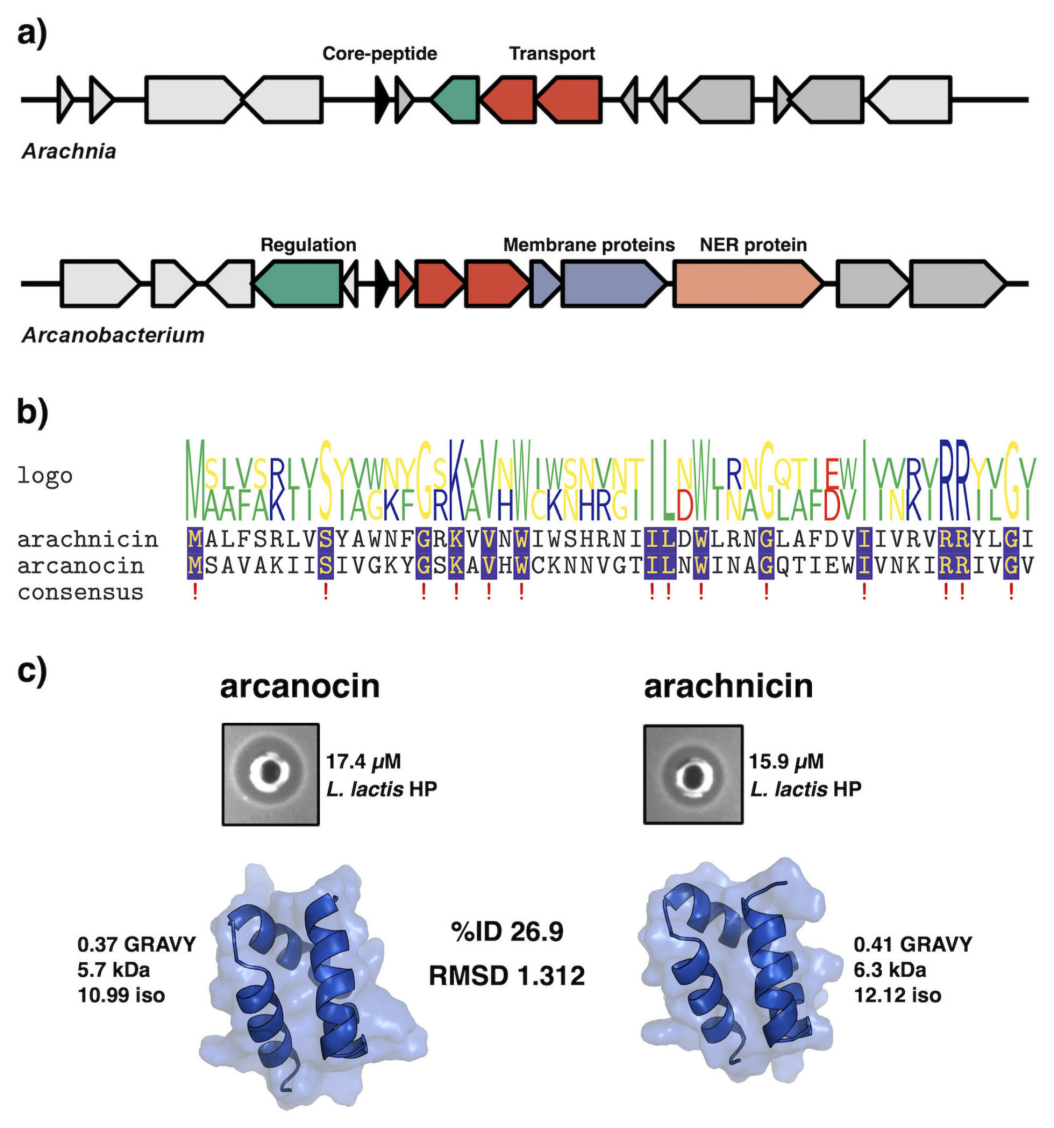
Characterization of two novel leaderless bacteriocin BGCs encoded by *Arcanobacterium* spp. and *Arachnia* spp. with novel core peptides found in the MGnify database. (a) BGCs do not share a high degree of synteny. Black arrows are the core peptides, red arrows are transport machinery, green arrows are regulatory proteins, and blue is a PH domain-containing protein. The blue arrow represents a bPH_2 domain-containing protein. The light orange arrow is a nucleotide excision repair (NER) protein that is a member of the excinuclease ABC subunit B responsible for recognizing damaged DNA. (b) A multiple sequence alignment of the two novel leaderless bacteriocins. MGYG000298963_01692 (arachnicin) is from *Arachnia*, and MGYG000000642_01040 (arcanocin) is from *Arcanobacterium*. Although the peptides differ in amino acid content (26.9%), they share 14 conserved residues, including two tryptophan residues. Both core peptides have three to four tryptophan residues, but only two are conserved in position. (c) The structure of leaderless bacteriocins from *Arachnia* and *Arcanobacterium* share a relatively low sequence identity of 26.9% but conforms to almost identical tertiary structures with an RMSD of 1.312. Arcanocin and arachnicin have antimicrobial activity against *L. lactis* HP at 100 μg/mL.

Two peptides not found in the nr database were chosen for synthesis as they were of human microbiome origin, and both have low percentage amino acid identity to known aureocin A53-like peptides, arachnicin from *Arachnia*, and arcanocin from *Arcanobacterium*. Arachnicin shares 38.46% ID to lacticin Q, and arcanocin shares 50.00% ID to lacticin Q. The operons encoding the leaderless bacteriocin BGCs from the genera *Arcanobacterium* (MGYG000000642) and *Aracnhia* (MGYG000298963) were found in gut and oral microbiomes, respectively. *Arcanobacterium* was assembled from a fecal stool sample from rural communities in north-eastern Madagascar (PRJNA485056), and *Aracnhia* was assembled from an oral microbiome assessing the clinical relevance of a dental implant microbiome (PRJEB43277). Both high-quality MAGs have over 96% completeness and less than 2% contamination, and both represent potential novel species within their genera. The operons found in both MAGs are less than 5-kb long but have a low degree of synteny and vary in gene content (Fig. 4a). The operon in *Arcanobacterium* sp. is longer and encodes seven genes putatively involved in the production of arcanocin. These include a PH domain-containing protein (dark blue), ABC transport machinery (red) and a putative bPH_2 domain-containing protein (light blue). The BGC from *Arcanobacterium* also has a longer contiguous operon with a putative PH domain-containing protein and a nucleotide excision repair protein that is not present in the *Arachnia* BGC. The BGC from *Arachnia* sp. has four genes associated with leaderless bacteriocin production, but the putative regulatory protein and transport system are on opposing strands to the core peptide. It is likely the operon is non-functional due to the organization of the operon, but it is possible that its non-contiguous nature could be a form of regulation (29, 30). Both short open reading frames beside each core peptide are hypothetical proteins. The BGCs encode single core peptides that are each 52 amino acids in length and share 14 residues (26.9% ID). Notably, the core peptides share tryptophan residues at positions 21 and 32 (Fig. 4b). The peptides also have three to four tryptophan residues in total, which probably play a role in their mechanism of action and are crucial for activity in some leaderless bacteriocin cases (15). Arachnicin (MGYG000298963_01692) has 38.5% ID to lacticin Q, and arcanocin (MGYG000000642_01040) has 53.8% ID to lacticin Z. Arcanocin and arachnicin are structurally identical with a root mean square deviation (RMSD) < 1.312 (Fig. 4c). The peptides have different physiochemical profiles. Arachnicin is more hydrophobic (0.41 compared to 0.37 GRAVY index), higher in molecular weight (6.3 kDa compared to 5.7 kDa), and has a much higher isoelectric point compared to arcanocin (12.12 compared to 10.99) (Fig. 2b). Both peptides were chemically synthesized. Synthesized arcanocin was soluble in H_2_O + 0.1 trifluoroacetic acid (TFA) at 100 μg/mL, whereas synthesized arachnicin was insoluble and had to be resuspended in acetonitrile (ACN) + 0.1% TFA. Both peptides were active against *L. lactis* HP at a concentration of 100 μg/mL (15.9 μM and 17.4 μM for arachnicin and arcanocin, respectively; Fig. 4c).

## Discussion

In this study, we identified 757 aureocin A53-like bacteriocins from the nr and MGnify databases, highlighting that they are more widespread than previously thought and are prevalent in the Actinomycetota. *Streptomyces* are one of the most productive bacteria in terms of biosynthetic potential, and recent focus has shifted to other members of the class Actinomycetes, such as *Nocardia* species, where some bacteria can have up to 70 BGCs (31). A total of 97 were identified within the phylum with a mean length of 55 amino acids. To date, no bacteriocins have been described in the genera *Arcanobacterium* and *Aracnhia*, nor has any leaderless bacteriocin-producing organism been characterized within the Actinomycetota. *Arcanobacterium* are facultative anaerobic gram-positive bacteria from the Actinomycetota. The genus currently has 63 species spread across the mammalian microbiota, with isolates from a harbor seal and rhinoceros (32, 33). The genus *Arcanobacterium pyogenes* was reclassified into the genus *Trueperella* in 2011, and this is now creating interest as a skin pathobiont named *Trueperella pyogenes*, which is a common cause of “summer mastitis” and pneumonia in bovine herds and pyometra in canines, respectively (34, 35). One of the most well-studied members of the genus is the rare pathogen *Arcanobacterium haemolyticum*, which is a facultative anaerobic bacterium previously classified as a *Corynebacterium* sp. (36).

The peptides arcanocin and arachnicin were chosen for gene synthesis based on low % ID to characterized leaderless bacteriocins and that they were found in MAGs assembled from the human gut and oral microbiome, respectively. They were also not present in the nr database at the time of synthesis, and they are from genera without characterized bacteriocin production. Arachnicin is most similar to lacticin Q (38.5% ID) and is one of the more hydrophobic peptides with one of the highest isoelectric points in the data set, and the sequence has two aspartate residues with acidic side chains. The peptide was not soluble in H_2_O + 0.1% TFA alone, likely because of these properties. Due to this, the peptide was resuspended in ACN + 0.1% TFA and presented antimicrobial activity against *L. lactis* HP at 100 μg/mL. TFA is a widely used chemical solvent used in the purification of peptides and has been used to solubilize garvicin KS and micrococcin P1 (37, 38). It is an effective solvent of hydrophobic peptides (39). However, the solvent is toxic to eukaryotic cells and is considered a contaminant during downstream purification of proteins after reversed-phase high-performance liquid chromatography (40). The removal of TFA as a solvent while retaining antimicrobial activity would be required if arachnicin was to be considered as an antimicrobial agent. TFA has been replaced with HCl during purification of pediocin PA-1 and the end product retained equal activity (39). Similarly, the bacteriocin nisin has 10-fold increased solubility at pH 3.0 compared to pH 7.0, and bio-engineered variants of the peptide have increase solubility which suggests arachnicin could be engineered to improve overall solubility at a higher pH (41). *Arachnia* is a genus from the gram-positive family Propionibacteriaceae that produces the metabolic by-product propionic acid (42, 43). There are only two species within the genus, *Arachnia propionica* and *Arachnia rubra*, and both are members of the oral microbiome (44). Interestingly, this family contains *Cutibacterium acnes*, a commensal bacterium with a clade, clade A1, associated with acne vulgaris and a causative agent of contamination of medical devices (45). The arcanocin peptide shares 26.9% ID and has a highly similar tertiary structure (RMSD 1.312). The peptide has a much lower isoelectric point than arachnicin, was soluble in H_2_O + 0.1% TFA, and was effective against *L. lactis* HP at 100 μg/mL.

PH domain-containing proteins are commonly found within BGCs. These proteins are found in many domains of life, play a part in cellular trafficking in eukaryotic cells, and are lipid-binding. Recently, it has been shown that PH domain-containing proteins have a role in the immunity and transport of leaderless bacteriocins (46). Interestingly, the formation of oligomeric ring structures that may resemble membrane-bound transporters supports this evidence, with the PH domain likely playing a role in oligomerization (47). However, PH domain-containing proteins are not present in all leaderless bacteriocin BGCs, suggesting there are multiple mechanisms that can be involved in providing immunity and transport of the core peptide after translation, and these mechanisms could be unique to the sub-classification of leaderless bacteriocin. This can be seen with the operons encoding enterocin DD14 and enterocin Q, where the former encodes PH domain-containing proteins involved in transport, whereas the latter does not (14, 46, 48). Another observation is Yip1 domain-containing proteins within the BGCs, which can also be observed in circular bacteriocin BGCs (5). The Yip1 family of proteins are also involved in cellular trafficking within eukaryotes, and they possess multiple transmembrane domains potentially suggesting a role in immunity or transport (49). Leaderless bacteriocin BGCs do not have a “class defining protein” such as the DUF95 domain membrane protein of circular bacteriocins, nor do they have a highly conserved immunity mechanism. The peptides are also bioactive after translation and are usually encoded with different membrane-associated protein families, suggesting a convergent evolution of immunity systems to protect from post-translational cell suicide. A future method to find dissimilar peptides of novel classes could be to use Yip1 domain-containing proteins and PH domain-containing proteins as driver sequences, as seen with the DUF95/SpoIIM protein in circular bacteriocins (50).

One exploitable trait of leaderless bacteriocins is the ability to capitalize on their “active after translation” trait because the N-formylation of methionine is not crucial for the antimicrobial activity of leaderless bacteriocins, such as aureocin A53 and lacticin Q (10). This trait allows chemical synthesis without needing accessory genes to produce active peptides. This can be exploited by chemically synthesizing peptides found in MAGs, which can dramatically reduce costs associated with production and allow the analysis of bioactive peptides based on DNA sequence alone.

This work identified 757 aureocin A53-like leaderless bacteriocins, 97 of which are in the phylum Actinomycetota, highlighting that they are present beyond the Bacillota. Chemically synthesized arcanocin and arachnicin, two novel leaderless bacteriocins from *Arcanobacterium* and *Arachnia*, respectively, are the first leaderless bacteriocins from the phylum Actinomycetota with demonstrated antibacterial activity. Actinomycetota is a phylum with a higher percentage of hypothetical proteins within their genomes than the Bacillota (51). This highlights the potential of looking beyond the Bacillota for novel bacteriocin production.

## Materials And Methods

### Genome mining

The nr and MGnify databases were downloaded in FASTA format in November 2023. MGnify protein databases included human gut, human oral, human vaginal, bee gut, rumen, pig gut, chicken gut, fish gut, marine, and zebrafish gut. MGnify databases were reduced to include proteins under 100 residues only using Seqkit v2.3.0 and the command “seqkit seq -M 100” (52). A leaderless bacteriocin HMM was created with known aureocin A53-like bacteriocins by aligning sequences with muscle v3.8.31 (53), and a model was created with the sequence alignment using HMMER v3.3.1 with the command “hmmbuild” (54). This model was used to search the MGnify protein database, and these were added to create a second model that was then searched back into both databases. All hits below an *e*-value of 1^e−5^ across the whole protein were brought forward as putative leaderless bacteriocins. Rodeo2 v2.3.3 was used to retrieve contig accessions, and assembly accessions were retrieved using Biopython v1.81 (55).

### Phylogenetic tree of organisms

Whole genome assemblies were downloaded using nbci-genome-download v0.3.3 (https://github.com/kblin/ncbi-genome-download). GTDB-Tk v2.3.1 was used with the commands “identify, align, infer” without the GTDB data included to create a *de novo* phylogenetic tree (56). A tree was created using FastTree and visualized using ggtree v3.18. Taxonomy was assigned to whole genomes using GTDB-Tk “classify --skip_ani_screen” using pplacer v1.1.alpha19-0-g807f6f3.

### Sequence similarity network

Core peptides were identified from the “.faa” files generated by Bakta by using the previously mentioned leaderless bacteriocin HMM using the command “hmmsearch --tblout.” These sequences were then retrieved using the command “grep -hA 1” under the rule get_proteins of the Snakemake workflow and saved as “final_proteins.faa.” This file was then uploaded to the Enzyme Function Initiative-The Enzyme Similarity Tool (EFI-EST) (57). A sequence similarity network (SSN) was constructed of the core peptides only using EFI-EST with a blast value of 1^e−05^. The corresponding network was visualized in Cytoscape v3.10.1 with the “prefuse force-directed” layout, and nodes were colored based on the GTDB phyla.

### Phylogenetic tree of peptides

Sequences were clustered at 90% identity using CD-HIT v4.8.1, and sequences were aligned using muscle v3.8.31. RAxML-NG v1.2.0 was used to construct a phylogenetic tree with the options “--model LG, --bs-trees 1000 --bs-metric tbe,fbp” (58). The tree includes the sequences for pediocin PA-1, enterocin A, enterocin CRL35, and sakacin *P* and was rooted to pediocin PA-1 using the R package ape v5.8. The tree was plotted using ggtree v3.18 (59).

### Peptide properties

Core peptide properties were predicted using the R package Peptides v2.4.6 (https://cran.r-project.org/web/packages/Peptides/index.html). The properties chosen were hydrophobicity, isoelectric point, molecular weight, and charge. Hydrophobicity was calculated using the GRAVY hydrophobicity using the “KyteDoolittle” scale. The isoelectric point was calculated using the “EMBOSS” pKscale. Properties were plotted using the ggplot2 v3.4.4. R package and R v4.3.0. Protein structures were predicted using Alphafold v2.3.2 with the setting “monomer” (60). A % ID matrix was created using predicted proteins and known leaderless bacteriocins using clustalO v1.2.4 and the parameters “--distmat-out --threads 12 --percent-id --full”. This was then used to compare predicted leaderless bacteriocins to known peptides. Only distinct core peptides were brought forward to calculate differences between aureocin A53-like peptides from Actinomycetota and Bacillota. The Kruskal–Wallis test was used to determine the statistical significance between the two groups using the stats v3.6.2 R package.

### Synteny plots

Assemblies were annotated using Bakta v1.8.1 with the Bakta database v5.0 (61). Operons were plotted using gggenomes v0.9.9.9000 and R v4.3.

### Peptide synthesis

Peptides arcanocin and arachnicin were synthesized by ProteoGenix (Schiltigheim, France). Arcanocin was resuspended in H_2_O + 0.1% TFA to a final concentration of 100 μg/mL. Arachnicin was resuspended in ACN + 0.1% TFA.

### Well diffusion assay

*L. lactis* HP was grown overnight using GM17 media at 30°C. Agar (0.75% wt/vol) was seeded with 0.2% (vol/vol) culture. The wells were created using a sterile Pasteur pipette. Fifty microliters of crude peptide solution (100 μg/mL) was added to the well and incubated at 30°C overnight. The antimicrobial activity was determined by the presence of an inhibition halo. The results were obtained from two independent replicates.

## Supplementary Material

Supplementary Figure Files

Supplementary Table S1

Supplementary Table S2

Supplementary Table S3

Supplementary Table S4

## Data Availability

Scripts and methods used in this study are stored in https://github.com/DEHourigan/leaderless_project

## Importance

Bacteriocins are gathering attention as alternatives to current antibiotics given the increasing incidence of antimicrobial resistance. Leaderless bacteriocins are considered a commercially attractive subclass of bacteriocins due to the ability to synthesize active peptide and low levels of documented resistance. Therefore, in this work, we mined publicly available data to determine how widespread and diverse leaderless bacteriocins are within the domain of bacteria. Actinomycetota, known for its antibiotic producers but lacking described and characterized bacteriocins, proved to be a rich source of leaderless bacteriocins—97 in total. Two such peptides, arcanocin and arachnicin, were chemically synthesized and have antimicrobial activity. These bacteriocins may provide a novel source of novel antimicrobials that could aid in the development of future alternative antimicrobials and highlight that the Actinomycetota are an underexplored resource of bacteriocin peptides.
