# Supplementary material for: Discovery and synthesis of leaderless bacteriocins from the Actinomycetota
Source: J Bacteriol. 2024 Oct 15;206(11):e00298-24. doi: 10.1128/jb.00298-24 (PMC11580447; doi:10.1128/jb.00298-24)
Supplement: Supplementary file 1 [file jb.00298-24-s0001.pdf]

Discovery and synthesis of leaderless bacteriocins from the Actinomycetota; Supplementary Figure File.

David Hourigan[1,2], Felipe Miceli de Farias[1], Paula M. O'Connor[1,3], Colin Hill[1,2], Paul Ross[1,2,3]

[1] APC Microbiome Ireland, Biosciences Institute, Biosciences Research Institute, College Rd, University College, Cork, Ireland

[2] School of Microbiology, University College Cork, College Rd, University College, Cork, Ireland

[3] Teagasc Food Research Centre, Moorepark, Moorepark West, Fermoy, Co. Cork, Ireland

Correspondence: p.ross@ucc.ie

logo

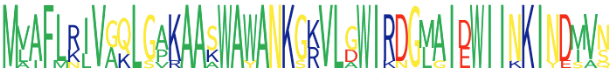

```
DEKJBI 27400 B. mycoides                           MVAFLRIVGQLGAKAASWAWANKGRVLDWIRDGMAIEWIINKINDMVS
EKFLIJ 02795 B. weirdmanii                         MVAFLRIVGQLGAKAASWAWANKGRVLGWIRDGMAIEWIINKINDMVN
DEKJBI 27405 B. mycoides, B. weirdmanii            MVAFLRIVGQLGAKAASWAWANKGKVLDWIKNGMAIDWIINKINDMVN
DEKJBI 27375 B. mycoides (5), B. weirdmanii (4)    MVAFLRIVGQLGAKAASWAWANKGKVLGWIRDGLAIDWIINKINDMVS
EAHPIB 01900 B. anthracis (4)                      MLAFLKLVAKLGPKAAKWAWANKGKVLGWIRDGLAIDWIINKINDIVN
EAHPIB 01920 B. anthracis                          MLAFLKLVAKLGPKAAKWAWANKSRVLGWIRDGMAIEWIINKINDIVN
DEKJBI 27370 B. mycoides, B. weirdmanii            MAIFMNIVAQLSVRAAAWAYANKGKVLAWIRDGLGIDWIIKKIYESAQ
consensus                                          !*!!!**!**!!*!!!*!!!!!!!*!!*!!!!!*!!*!!!!!!!!*!*
```

X non-conserved

X $\geq 50\%$ conserved

X $\geq 90\%$ conserved

Fig. S1. Multiple sequence alignment of core peptides from *B. mycoides*.

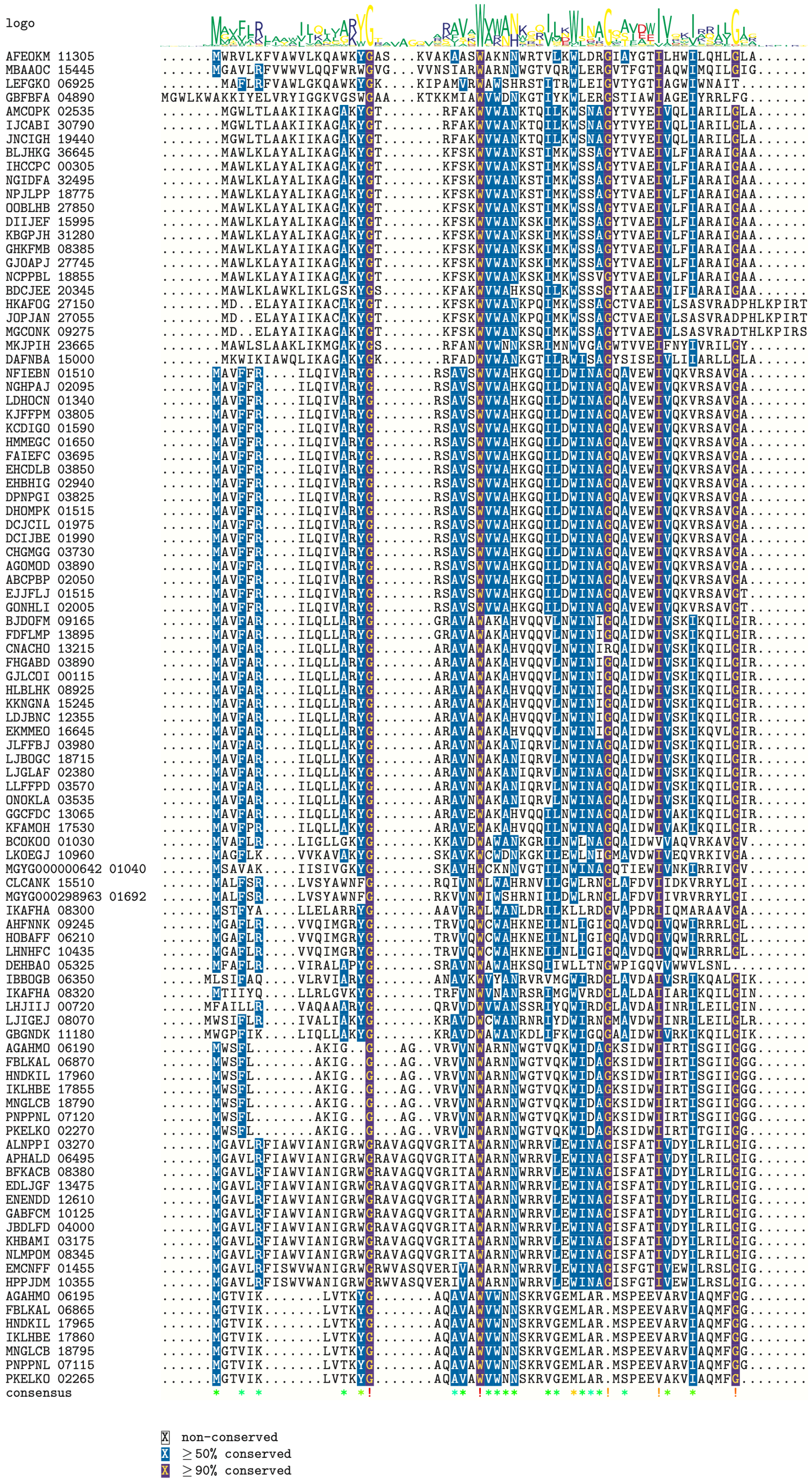

Fig. S2. Multiple sequence alignment of core peptides discovered from the phylum Actinomycetota.

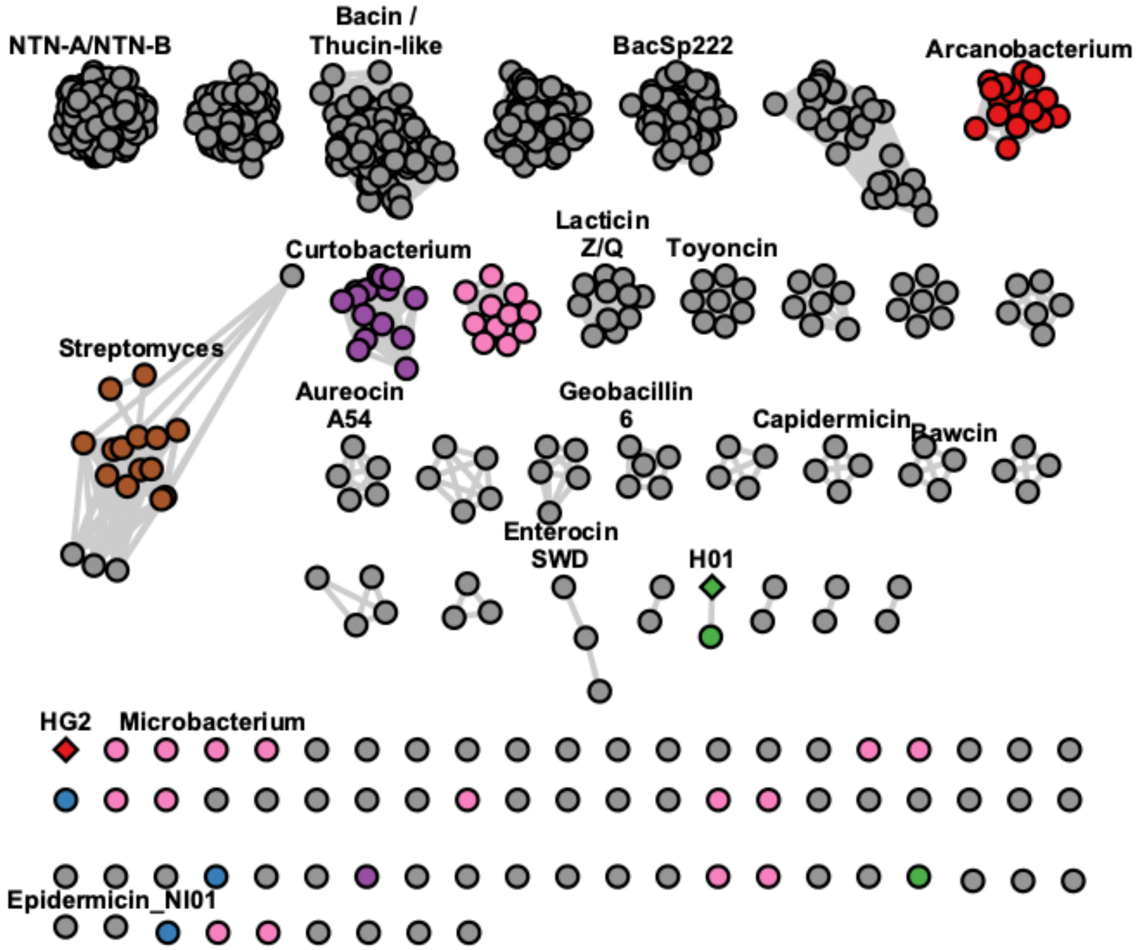

Fig. S3. SSN coloured by the top four genera that are part of the phylum Actinomycetota. Red represents peptides from the genus *Arcanobacterium*. Brown represents peptides from *Streptomyces*. Purple are peptides found in *Curtobacterium* and pink are peptides from *Microbacterium*. Peptides in blue are from *Bifidobacterium* and green are from *Arachnia*.

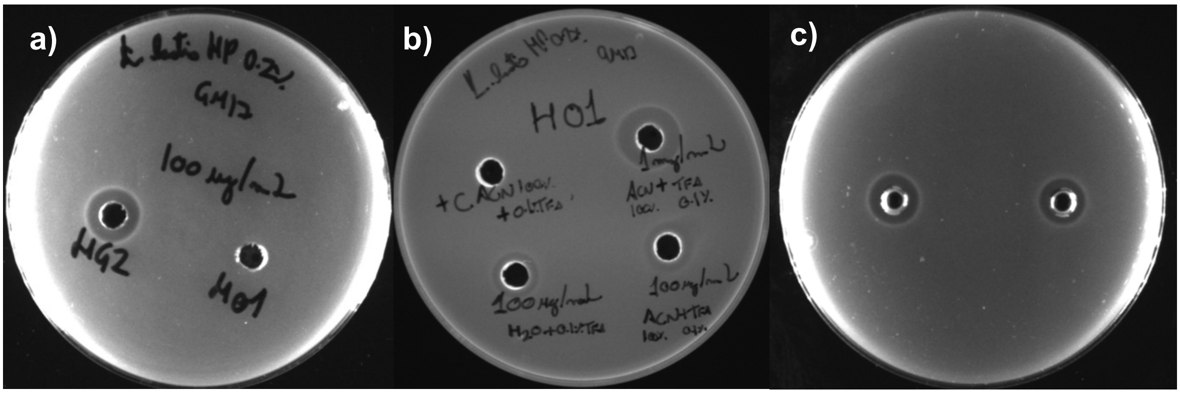

Fig. S4. Antimicrobial activity of arcanocin from *Arcanobacterium* sp. and arachnicin from *Arachnia* sp. against *Lactococcus lactis* HP. (a) Antimicrobial activity of arcanocin resuspended in H_2O at a final concentration of 100 ug/mL against *L. lactis* HP. Arachnicin was not soluble in H_2O and showed no antimicrobial activity in H_2O at 100 ug/mL. **(b)** Arachnicin solubilised in Acetonitrile (Acn) and 0.1% Trifluoroacetic acid (TFA) at 1mg/ml (top right well), 100 ug/ml (bottom right well). Acn and TFA control well (top left) and activity of arachnicin in 100 ug/ml suspended in H_2O plus 0.1% TFA. **(c)** The left well is 100 ug/mL arcanocin and the right well is 100 ug/mL of arachnicin against *L. lactis* HP. Both wells have 50uL added to them.

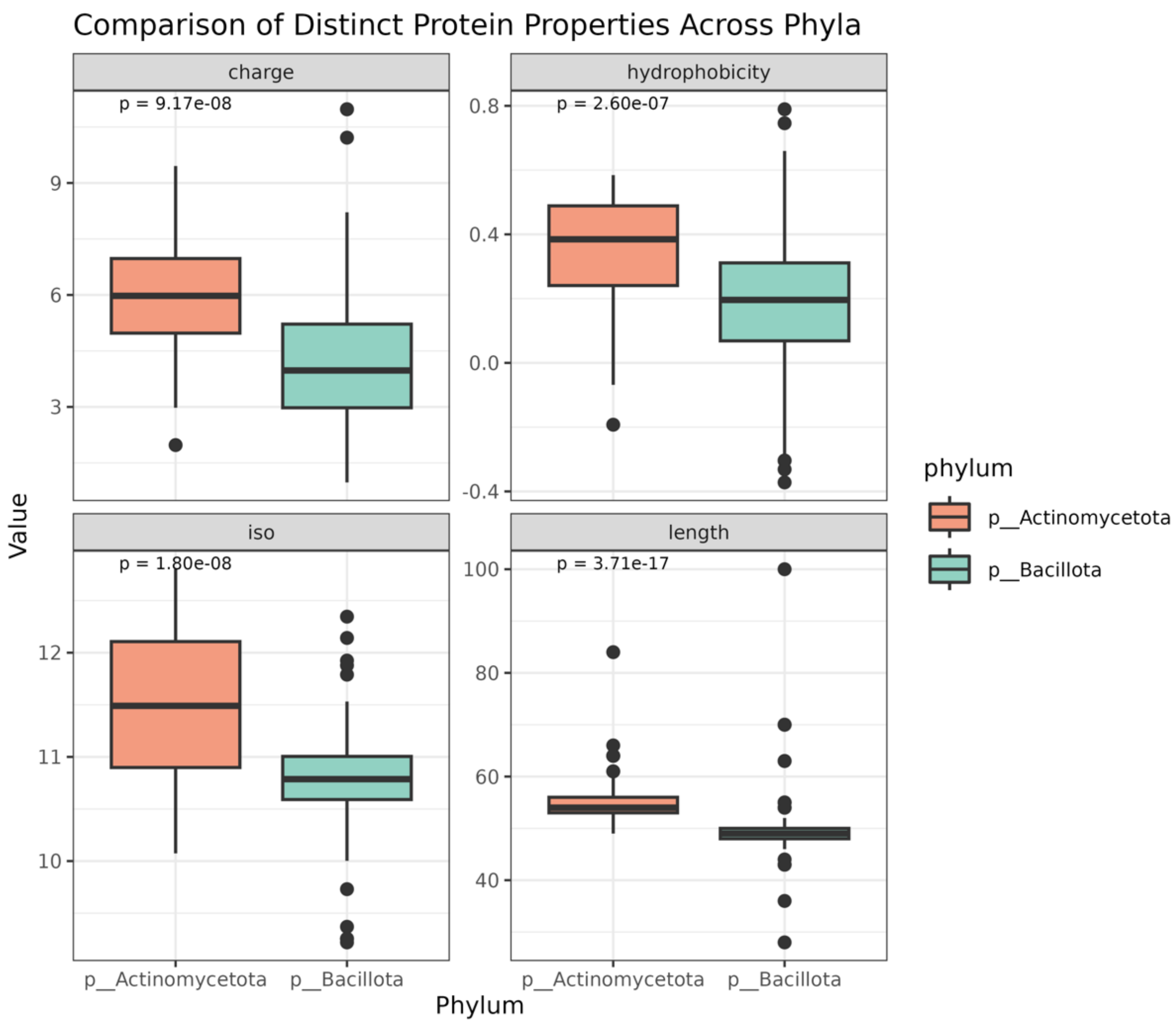

Fig. S5. Comparison of properties between leaderless bacteriocins from the phyla Actinomycetota and Bacillota. The data set was reduced to only unique core peptides to remove the bias towards more frequently found peptides. The Kruskal-Wallis test was used to determine the statistical significance between the two groups. Isoelectric point is labelled as “iso”.

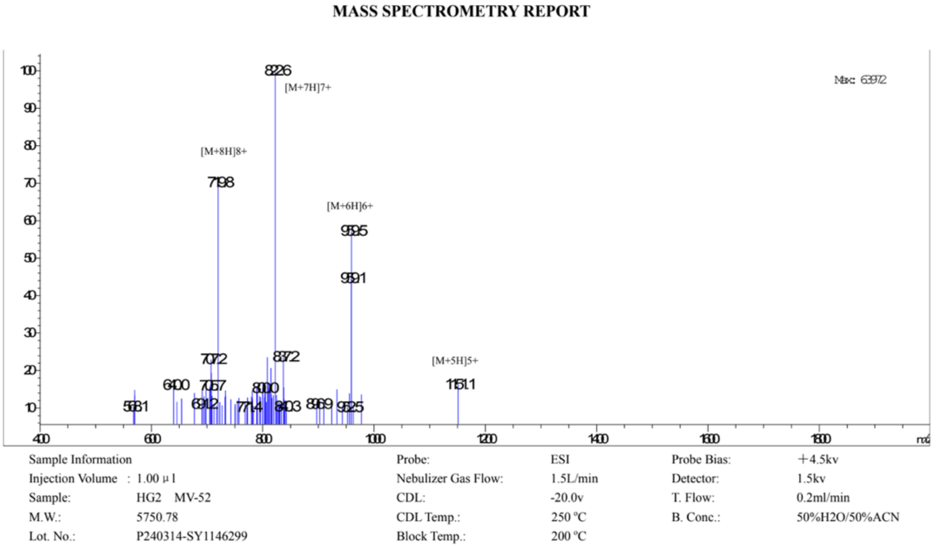

Fig. S6. Mass spectrometry report for peptide arcanocin.

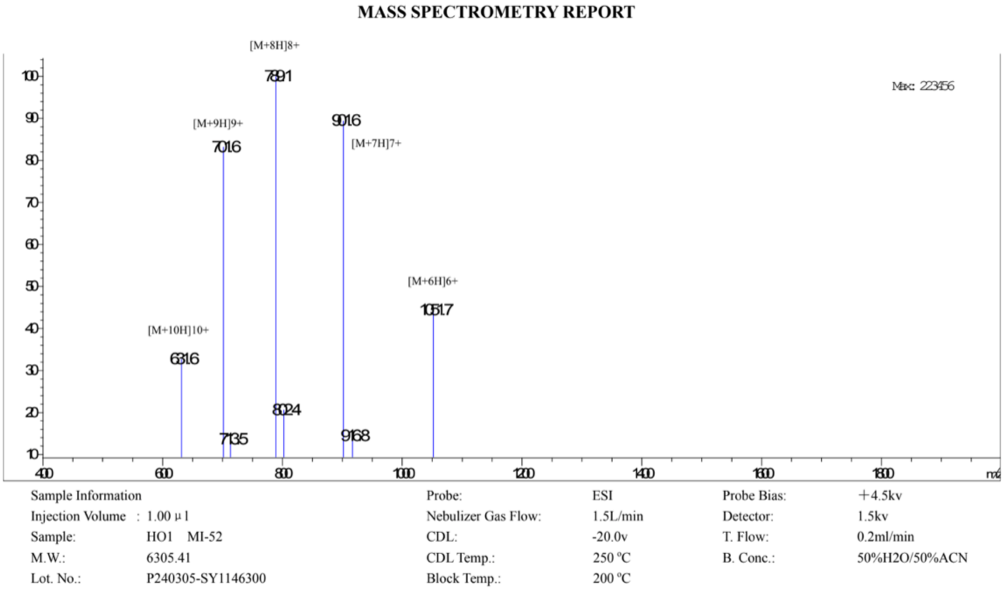

Fig. S7. Mass spectrometry report for peptide arachnicin.

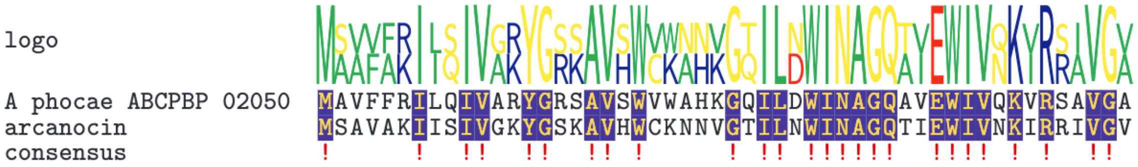

Fig. S8 Sequence alignment of arcanocin and a representative from the cluster of peptides from the species *Arcanobacterium phocae*.

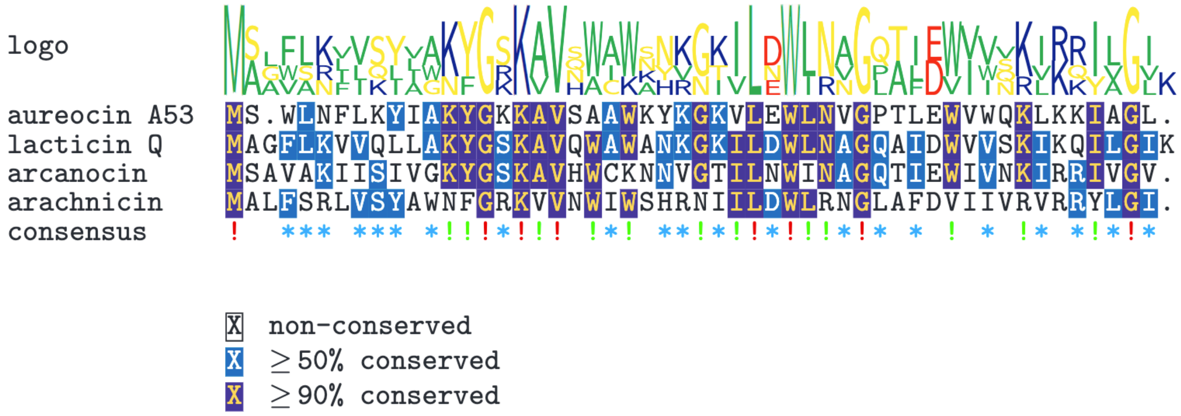

Fig. S9 Sequence alignment of arcanocin, arachnicin, aureocin A53 and lacticin Q.
